# Extracting decision-making features from the unstructured eye movements of clinicians on glaucoma OCT reports and developing AI models to classify expertise

**DOI:** 10.3389/fmed.2023.1251183

**Published:** 2023-09-29

**Authors:** Michelle Akerman, Sanmati Choudhary, Jeffrey M. Liebmann, George A. Cioffi, Royce W. S. Chen, Kaveri A. Thakoor

**Affiliations:** ^1^Department of Biomedical Engineering, Columbia University, New York, NY, United States; ^2^Department of Computer Science, Columbia University, New York, NY, United States; ^3^Edward S. Harkness Eye Institute, Department of Ophthalmology, Columbia University Irving Medical Center, New York, NY, United States

**Keywords:** eye-tracking, fixations, optical coherence tomography, glaucoma, unsupervised clustering, neural networks

## Abstract

This study aimed to investigate the eye movement patterns of ophthalmologists with varying expertise levels during the assessment of optical coherence tomography (OCT) reports for glaucoma detection. Objectives included evaluating eye gaze metrics and patterns as a function of ophthalmic education, deriving novel features from eye-tracking, and developing binary classification models for disease detection and expertise differentiation. Thirteen ophthalmology residents, fellows, and clinicians specializing in glaucoma participated in the study. Junior residents had less than 1 year of experience, while senior residents had 2–3 years of experience. The expert group consisted of fellows and faculty with over 3 to 30+ years of experience. Each participant was presented with a set of 20 Topcon OCT reports (10 healthy and 10 glaucomatous) and was asked to determine the presence or absence of glaucoma and rate their confidence of diagnosis. The eye movements of each participant were recorded as they diagnosed the reports using a Pupil Labs Core eye tracker. Expert ophthalmologists exhibited more refined and focused eye fixations, particularly on specific regions of the OCT reports, such as the retinal nerve fiber layer (RNFL) probability map and circumpapillary RNFL b-scan. The binary classification models developed using the derived features demonstrated high accuracy up to 94.0% in differentiating between expert and novice clinicians. The derived features and trained binary classification models hold promise for improving the accuracy of glaucoma detection and distinguishing between expert and novice ophthalmologists. These findings have implications for enhancing ophthalmic education and for the development of effective diagnostic tools.

## 1. Introduction

With an estimated 60.5 million people worldwide diagnosed with glaucoma in 2010, glaucoma stands as the leading cause of irreversible blindness globally (1). As populations age worldwide, future burden of disease detection, monitoring and treatment will necessitate the development of more effective tools and strategies to address the impact of this disease on diverse populations (1). Additionally, there is less consensus on reference standards for the diagnosis of early disease, unlike other eye diseases such as age-related macular degeneration (2) and diabetic retinopathy (3). This makes it challenging to synthesize multiple ophthalmic test results and draw precise conclusions for diagnosis (4). Optical coherence tomography (OCT) is increasingly becoming a primary tool for detecting the presence or absence of glaucoma. However, there are few universally agreed-upon diagnostic features in OCT reports even among clinicians; thus, evaluating such reports to diagnose glaucoma requires a significant level of expertise (4). Given the challenges in diagnosing glaucoma and the global and local costs of care and vision-related disability, artificial intelligence (AI) and machine learning (ML) can serve as valuable tools to expedite accurate eye disease screening and diagnosis.

The critical role of AI and ML is highly evident in the field of medicine given the massive amounts of available data. These intelligent systems can promote or assist accurate image interpretation at the single-clinician level and can also improve clinic-wide support for the disease diagnosis workflow (5). Physicians are constantly accumulating multisensory evidence when inspecting images and patient information to arrive at diagnostic decisions (6). Specific to this current manuscript, eye-tracking research allows for an increased understanding of the dynamics involved in the human (medical-expert’s) interpretative processes. This includes identifying qualitative and quantitative differences that can assist in distinguishing between successes and errors. Such advancements provide valuable perspectives on how to enhance and maintain the interpretative process during education/training and may even inform the role of AI in clinical practice (6).

Studies in the medical domain using eye trackers have provided a more nuanced understanding of the diagnostic decision-making process in fields such as radiology, pathology, pediatrics, surgery, and emergency medicine (7–9). In the past, advanced clinical tutorials for studying laparoscopic surgery (10) and for the management of ‘deteriorating patients’ (11) were developed through the application of eye-tracking technology on expert and novice clinicians. Some studies have also performed automatic detection of interpretative errors (in physician eye movements) that arise during diagnostic procedures (12) ranging from mammography screening to various radiological assessments (13, 14). These examples show the potential for eye tracking to revolutionize clinical practice and medical education.

In the realm of ophthalmology, eye-tracking technologies have been employed to evaluate the eye gaze patterns of ophthalmologists while examining retinal fundus images with diabetic retinopathy (15). One study compared the gaze patterns and search strategies of expert ophthalmologists and trainee ophthalmologists as they assessed and classified fundus images (16). The study’s findings indicated that fully trained ophthalmologists had shorter interpretation time, a longer time to first fixation, decreased visit and fixation counts, and increased accuracy scores and confidence in diagnosis in comparison to ophthalmology trainees.

Within the realm of glaucoma research, previous studies have considered eye movements in patients with glaucoma compared to visually healthy controls. Eye tracking has been used as a tool to evaluate functional ability in everyday tasks (17) as well as reading performance (18) of glaucoma patients with impaired visual search. The results of these studies provide meaningful insight into the functional deficits of glaucoma (19) through detection of fixations and saccades.

Our own past work was one of the first studies (4) to compare eye movement regions fixated most by glaucoma experts with regions of importance as determined by AI systems; here we build on that work by evaluating gaze patterns and differences in eye gaze metrics of ophthalmologists with varying training experiences, as they evaluated and classified OCT reports for the detection of glaucoma. This study has three main aims. First, we evaluate how eye gaze metrics and gaze patterns evolve as ophthalmic education increases. Second, we derive new features from eye-tracking data that can shed light on the search strategies of experts while they scan full OCT reports to determine the presence of glaucoma. Third, we extract these key features to develop binary neural network classification models that can distinguish between diseased and healthy images as well as between expert and novice training levels.

## 2. Materials and methods

### 2.1. Experimental data collection

Thirteen ophthalmology residents, fellows, and clinicians (glaucoma specialists) at Edward S. Harkness Eye Institute, Department of Ophthalmology, Columbia University Irving Medical Center (CUIMC), were recruited to the study. The participants were grouped according to their level of experience (refer to Table 1) into three groups: junior (jr.) residents, senior (sr.) residents, and experts. The four ***jr. residents*** have less than 1 year of experience while the four ***sr. residents*** have 2–3 years of experience. The ***expert*** group consists of five fellow and faculty clinicians with over 3 years (and up to 30+ years) of experience. Each participant was shown a set of 20 Topcon swept-source OCT (Triton, Topcon, Inc., Paramus, NJ, United States) reports and was asked to identify the absence or presence of glaucoma and rate their confidence of diagnosis on a scale from 0 to 100, where 0 corresponds to ‘Definitely Not Glaucoma’ and 100 corresponds to ‘Definitely Glaucoma’. After evaluating each report, participants were asked to indicate the specific features or regions of the report they considered and used when making the diagnosis. The set of OCT reports consisted of 10 healthy and 10 glaucomatous eyes presented in random order. Participants 1 through 7 evaluated a randomized set of 20 digital reports (from a superset of 55 de-identified Topcon OCT reports collected from patients who visited CUIMC between 2010 and 2023) with some overlap, and the remaining participants (8 through 15) assessed a controlled set of the same 20 digital OCT reports each, with complete overlap. The reports were presented in their original format and resolution (2015 × 3365 pixels), as shown in Supplementary Figure S1, just as they would be viewed by residents/experts in the clinic, without the option to zoom in on specific regions. Two clinicians were excluded from the study, one due to not being a resident and the other due to calibration error during data collection. We built our user-interactive experiment with the features described above using PsychoPy (Open Science Tools, Ltd., Nottingham, United Kingdom) and utilized Pavlovia (Open Science Tools, Ltd., Nottingham, United Kingdom) to host each session of the experiment online.

**Table 1:** Participant training levels.

Subject	EEM1	EEM3	EEM5	EEM6	EEM9	EEM7	EEM12	EEM4	EEM8	EEM10	EEM11	EEM13	EEM15
Training	10 months	10 months	10 months	11 months	23 months	23 months	35 months	36 months	Glaucoma Fellow	Glaucoma Faculty	Glaucoma Faculty	Glaucoma Faculty	Glaucoma Faculty
Group	Jr. residents				Sr. residents				Experts				
Label	Novice								Expert				

EEM2 and EEM14 were excluded due to non-resident status and data collection issues, respectively.

To collect eye movement data from the participants, each clinician was instructed to wear the Pupil Labs Core eye tracker (Pupil Labs GmbH, Berlin, Germany), a lightweight, mobile eye-tracking headset (20) with a sampling rate of 200 Hz and 0.60 degrees accuracy (21). This headset is equipped with one scene camera and two infrared eye cameras for dark pupil detection (20). The scene camera captures the scene in front of the participant (the stimuli) while the eye cameras are directed at the participant’s eyes. The Pupil Capture software analyzes each frame from the eye cameras and creates a 3D model of eyeball position to determine pupil position. A 5-point screen-marker calibration was carried out on each participant before eye movement recordings were captured to map pupil to gaze position. We added four unique April Tag markers to the four corners of each OCT report to define a planar tracking surface. This allowed the eye tracker to normalize eye gaze positions with respect to these defined surface coordinates each time a report was shown to a clinician. After calibration and surface definition, we recorded each participants’ eye movements as they diagnosed 20 digital OCT reports. Another software, Pupil Player, was used to export the data from the recording. When exporting the data, the following plug-ins were applied: Blink Detector, Fixation Detector, Raw Data Exporter, and Surface Tracker. The fixation detector detects minimum fixations of 100 ms (21). For the purposes of this study, the key data analyzed were fixation identifications (IDs), normalized *x* and *y* coordinates, starting timestamps, and an indication of whether the gaze was on the surface or not.

### 2.2. Preprocessing of data

As previously described, the exported data provided important information, such as the fixation IDs, the normalized *x* and *y* coordinates, and an indication of whether the gaze was directed at the surface. We extracted the data recorded only during the ON-surface condition, while the participants were viewing the OCT reports only. This avoided the use of any gaze data that was recorded when the participants were either describing the features used for diagnosis or rating the confidence of their diagnosis on a scale of 0 to 100.

The Pupil Capture software also provides an ID number for each of the fixations that occur. A fixation refers to a momentary pause of eye gaze at a spatial location for a minimum amount of time (e.g., 200–250 milliseconds) (22). For instance, a single recording may include a total of 1970 fixations, each labeled from 1 to 1970. Every fixation ID is associated with normalized *x* and *y* coordinates which are positions of the pupil ranging between 0 and 1 scaled with respect to the height and width of the displayed OCT report. This normalization allows for consistent interpretation of eye movement data, regardless of the different sizes and resolutions of the eye tracker surface (OCT report) (23).

**Figure 1:**
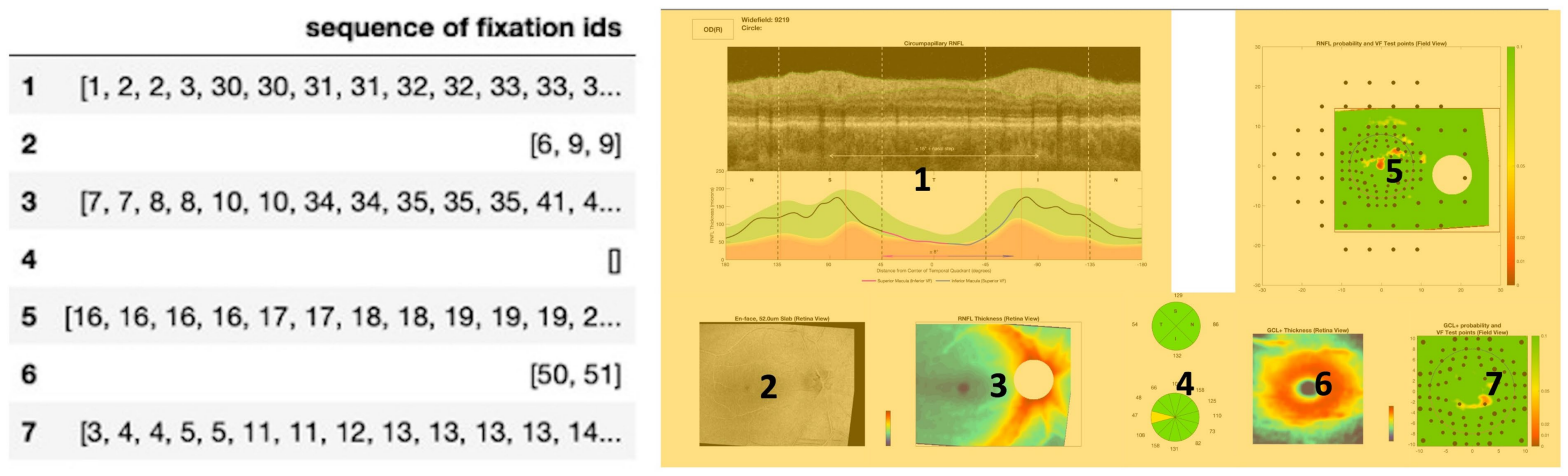
Left: Input structure for sequence graph transform (SGT) embedding that associates each fixation ID with a region of interest (ROI) from the OCT report. For example, the clinicians fixated on Region 1 (circumpapillary retinal nerve fiber layer (RNFL) b-scan) for IDs 1, 2, 3, 30, 31. Right: OCT report with the seven labeled regions of interest (ROIs), highlighted in yellow for enhanced contrast and visibility: Region 1 corresponds to the circumpapillary RNFL b-scan, Region 2 corresponds to the en-face slab, Region 3 corresponds to the RNFL thickness map, Region 4 corresponds to the sectoral thickness pie charts, Region 5 corresponds to the RNFL probability map, Region 6 corresponds to the retinal ganglion cell layer (GCL) + thickness map, Region 7 corresponds to the GCL + probability map.

We also defined seven regions of interest (ROIs) on the OCT report (Figure 1, right) labeled from 1 to 7. Each of these ROIs represents a unique diagram/feature of the OCT report that is used by experts in order to provide an accurate diagnosis. For example, Region 5 represents the retinal nerve fiber layer (RNFL) probability map while Region 1 represents the circumpapillary RNFL b-scan, both of which are critical diagrams for the diagnosis of glaucoma. Figure 1, right provides a mapping between each numbered region and the name of a specific diagram/feature on the OCT report. Establishing these ROIs allowed us to evaluate each clinician’s spatial allocation of attention over the OCT report (24–26). We encoded each of the normalized coordinates based on these ROIs so that each coordinate and its associated fixation ID were assigned a label from 1 to 7, partitioning the fixation IDs across the different ROIs.

### 2.3. Statistical analysis of fixation number across diagnoses and training levels and design of supervised deep learning model

As an initial step in the visualization process, we plotted the fixations of all participants onto their corresponding OCT images. We visually inspected and compared the images in common between participant groups of varying expertise levels and observed a qualitative reduction in fixations with increasing expertise. Subsequently, we conducted a quantitative assessment to determine the statistical significance of this observed difference.

#### 2.3.1. Fixations: quantitative correlation analysis

To first test for normality in our dataset, a one-sample Kolmogorov–Smirnov test was performed on the number of fixations on both healthy and glaucomatous OCT reports across participants in each expertise group. The tests rejected the null hypothesis at the 5% significance level, failing the normality test.

##### 2.3.1.1. Intra-group statistical testing: comparison of number of fixations and response time in healthy vs. glaucomatous OCT reports

Thus, for each of the three training level groups, non-parametric, two-sided Wilcoxon rank sum tests were performed to compare the distribution of the number of fixations made by each group on the healthy OCT reports and on the glaucomatous OCT reports. The same statistical analysis was performed on the response time per report within each group and diagnosis-type to determine if the two independent groups of observations came from populations of different distributions and to evaluate the possible correlation of response time with number of fixations.

##### 2.3.1.2. Inter-group statistical testing: comparison of number of fixations and response time in OCT reports by diagnosis

We performed group comparisons using the same method as described above to evaluate the similarity in the distribution of the number of fixations and the response time of the collected eye data for healthy and glaucomatous OCT reports across groups of varying expertise. We did this to determine if the number of fixations was significantly lower for the expert group compared to the novice groups in healthy and glaucomatous reports, separately. Initially, inter-group statistical testing was performed on common images across groups, to exclude image differences when evaluating the fixation number. However, given the limited participant data in the study, this filtering process was discarded to increase statistical power; thus, all samples within the same diagnosis and expertise group were concatenated.

##### 2.3.1.3. Intra-group statistical testing: comparison of number of fixations per region in OCT reports, glaucomatous vs. healthy (within group)

Using the seven regions of interest outlined in Figure 1 (right), we performed statistical testing using the Mann Whitney U test to establish what regions on the OCT reports demonstrated statistically significant differences in the number of fixations across expertise groups for healthy and glaucomatous reports. This may provide important spatial information critical to the expert search strategy and lacking in the novice gaze pattern at the time of diagnosis.

##### 2.3.1.4. Inter-group statistical testing: comparison of number of fixations per region in OCT reports, novice vs. expert group

We next proceeded to establish what regions on the OCT reports demonstrated statistically significant differences in the number of fixations across expertise groups for healthy and glaucomatous reports. The Mann Whitney U test was conducted once more to complete this statistical analysis and to further establish the gaze search pattern differences between groups of varying expertise.

#### 2.3.2. Supervised deep learning: fixation number derived approach

**Figure 2:**
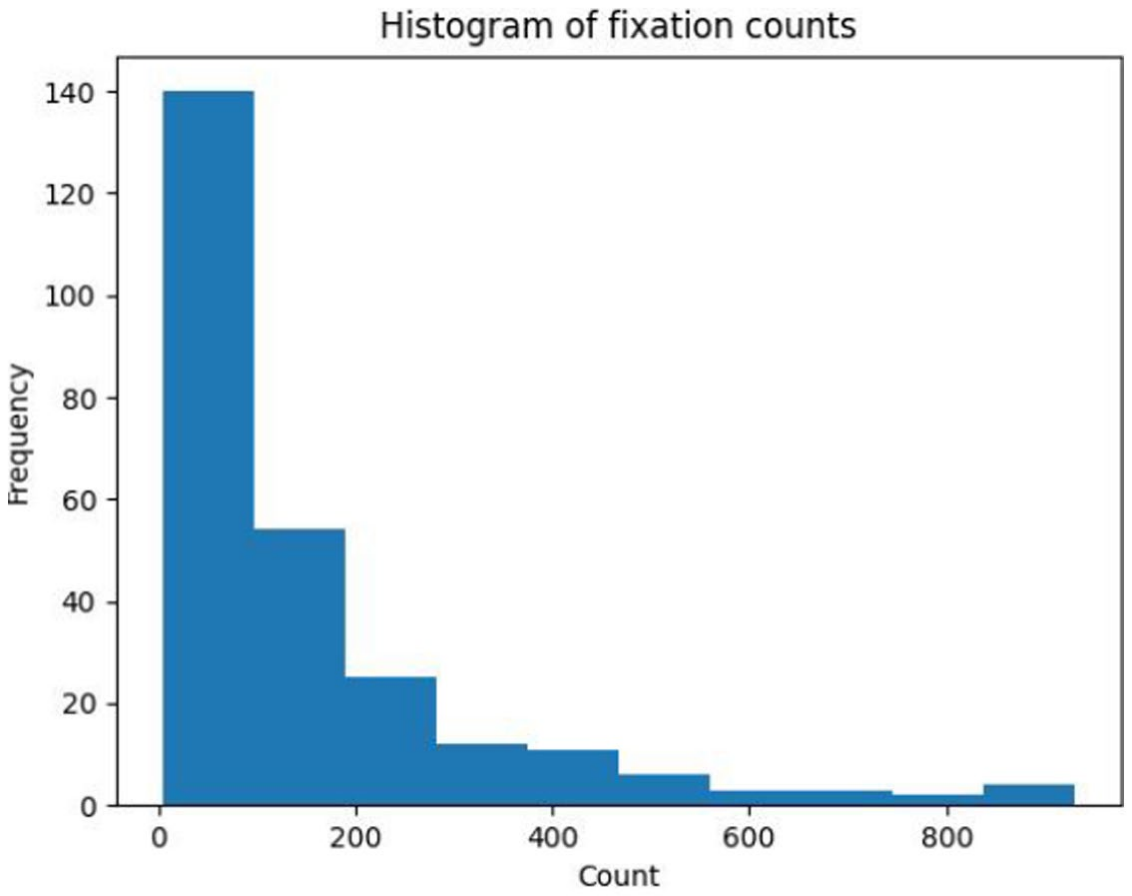
Histogram of fixation counts on OCT full reports read by participants.

An input feature vector based on the fixation trajectory of each subject for each presented OCT report was created, using the ROI encoding for the OCT report that was described earlier. The trajectory feature vector length was informed by the number of fixations across expertise groups and OCT reports (Figure 2), where the median fixation frequency was 85 fixations, the lower quartile was 37 fixations, and the higher quartile was 195 fixations. To ensure a consistent and manageable length for the trajectory feature vector, its length was set to 100 fixations. This choice strikes a balance between capturing sufficient information from eye movement patterns while avoiding excessive dimensionality that could potentially hinder the performance of subsequent analyses and models. All samples with longer feature vectors were cut to the first hundred fixations while samples with fewer feature vectors were zero-padded as needed to control for feature vector size.

The visual fixation dataset was split into training and testing data in an 80%:20% ratio. Two distinct classification tasks were performed using a simple neural network.

The neural network consisted of three fully connected layers, one rectified linear unit, one batch normalization layer, one dropout layer with a probability of 0.5, and one final sigmoid activation function. To standardize the input data, the mean was subtracted, and the result was divided by the standard deviation. The batch normalization layer after the first linear layer normalized the input to have zero mean and unit variance, which helped stabilize the training process and improved the performance of the model. The model was trained for with batch size of 8 and for 100 epochs, using the Adam optimizer, a learning rate of 0.0001, L1 regularization of 0.01, L2 regularization of 0.3, and binary cross-entropy with logit loss as its criterion.

The first classification task aimed to distinguish eye trajectories of expert participants when evaluating glaucoma versus healthy OCT reports. The model’s targets were defined as (0) for glaucoma and (1) for healthy labels. In the second classification task, the goal was to distinguish between eye trajectories of experts and novices (jr. and sr. residents) for both healthy and glaucoma reports. To achieve this, jr. and sr. residents were combined into a single “novice group,” which was assigned a label of 0, and compared to the “expert group” (composed of faculty and fellows), which was assigned a label of 1.

In the study, the model’s ability to discriminate between the two outcomes was quantified using two metrics: the train and test accuracy and the area under the curve (AUC) of the receiver operating characteristic (ROC). Train and test accuracy are calculated by dividing the number of correct predictions by the total number of predictions made on the training and test dataset, respectively. The AUC metric measures the model’s threshold-independent ability to distinguish between two outcomes, with a higher AUC indicating a better performance. The optimal threshold for model prediction was determined by maximizing Youden’s J Statistic on the curve, which equally weighs sensitivity and specificity to measure the effectiveness of a diagnostic algorithm, thereby helping to select an optimal threshold value for the algorithm of interest (27).

### 2.4. Mapping the relationship between fixations and developing unsupervised and supervised deep learning models

#### 2.4.1. Unsupervised learning

When analyzing eye-tracking data, it can be challenging to infer cognitive strategies from the locations of the participant’s gaze. In order to solve this task, eye movements can be grouped based on regions of interest (ROIs) in OCT reports that were fixated most in order to learn the presence or absence of common features and characteristics within varying expertise groups (28). For this study, we used ROIs as described above (and as shown in Figure 1, right). By detecting the qualitative differences between these fixated ROIs, we establish novel eye movement features informed by clinicians’ eye movements while diagnosing glaucoma. We used unsupervised methods to cluster similar eye fixation ROIs to gain insights into clinicians’ cognitive decision-making strategies (29, 30).

The fixations, as described above, were binned into predefined areas of interest (Figure 1, left). An input structure mapping each of the ROIs with its associated fixation IDs was created.

In order to make these data sequences machine-interpretable, a sequence graph transform (SGT) embedding was generated. This feature embedding uses similarity computations within the sequence in order to closely analyze the relationships between each of the fixation points for the specified regions of interest (31). The embedding is known to be robust to noise and effectively takes into account length sensitivity and insensitivity (31). In addition, for sequence clustering and classification, SGT achieves higher accuracy and requires less computation compared to existing methods such as kernels and Hidden Markov Models (31). The function returns a high-dimensional embedding that contains normalized similarity values between each of the fixations. Clustering methods generally take such vectors as input but due to the SGT embedding’s high dimensionality, Principal Component Analysis (PCA) was applied with two components. This allowed us to visualize and interpret the clusters in a two-dimensional space. SGT embeddings were generated for each OCT report for each of the thirteen clinicians. Clustering was applied to each of these embeddings in order to understand which regions of interest were being grouped together across each of the OCT reports.

#### 2.4.2. Supervised learning: embedding derived approach

The SGT embedding layer can also be used for supervised machine-learning tasks. These embeddings provide distinct information on regions of interest that can be used as inputs for deep learning binary classification tasks. The multi-dimensional SGT embedding was reduced to one principal component that represented the extent of similarity between the fixations in a given ROI. A 7-dimensional input vector was created that concatenated each of the dimensionality-reduced embeddings for all 7 ROIs. In this input structure, there are 260 rows for each of the 20 reports seen by the thirteen clinicians. There are also seven features labeled from 1 to 7 which represent the seven ROIs on the OCT scan (Figure 1, right). After generating this input structure, two supervised learning tasks were performed: predicting if the embedding represents a (1) healthy versus glaucomatous diagnosis or (2) a novice versus expert’s eye movements.

##### 2.4.2.1. Supervised task 1: predicting diagnosis

Healthy (1) and glaucomatous (0) labels were added to the input structure in order to denote whether the embedding generated for a specific OCT was healthy or glaucomatous. In addition, scaling and normalization were also applied in order to avoid numerical instability and make the features more comparable. For the classification task of healthy vs. glaucomatous prediction, clinician data from only **experts** (faculty/fellow) were used. There were five participants in this expertise category, and each diagnosed 20 reports which provided an overall sample count of 100 embeddings.

The SGT-embedding-generated data was inputted into a feed-forward neural network model, and this dataset was divided into training and testing sets using an 80% training to 20% testing ratio, repeated across 50 different, randomized train-test folds. The neural network consisted of four dense layers, one output sigmoid activation layer, and 2 dropout layers with a dropout probability of 0.1 in order to avoid overfitting. The model was trained with the Adam Optimizer for 20 epochs after which improvement in the accuracy of the prediction and loss values saturated.

##### 2.4.2.2. Supervised task 2: predicting expertise group

The second task of supervised learning consisted of predicting if the eye movements were from an expert (faculty/fellow) or from a novice (jr. and sr. residents). Labels of expert and novice were provided to each of the 260 samples in the input structure. Out of the thirteen participants, there were five experts and eight novices. Hence, there was a class imbalance of 100 expert samples and 160 novice samples in the data.

A feed-forward neural network model was trained on 80% of the data and the remaining 20% of the samples were used for testing across 50 distinct folds; the average test accuracy of all 50 train-test folds is reported. This model consisted of 3 dense layers with 2 dropout layers with dropout probability of 0.1 and 0.2, respectively. The model was trained with an Adam optimizer for 100 epochs.

## 3. Results

### 3.1. Statistical analysis of fixation number across diagnoses and training levels and design of supervised deep learning model

#### 3.1.1. Fixations quantitative correlation analysis

##### 3.1.1.1. Intra-group statistical testing: comparison of number of fixations and response time in healthy vs. glaucoma OCT reports

The Wilcoxon Rank Sum test reported no significant differences in the number of fixations for healthy and glaucomatous OCT reports within the **novice** groups (jr. resident: EEM1, EEM3, EEM5, and EEM6; sr. resident: EEM4, EEM7, EEM9, and EEM12). Within the **expert** group, the mean number of fixations detected on OCT reports of 92 fixations for the glaucomatous OCT reports was statistically significantly higher than the number of fixations detected on healthy OCT reports of 59 fixations, with a value of *p* of 0.00916 at the 5% significance level. Similarly, the mean response time for the glaucomatous reports of 13.98 s, was significantly greater than the mean response time for the healthy reports of 8.187 s, with a value of *p* of 0.00366. In other words, the participants in the expert group made significantly more visual fixations and took a significantly longer time to evaluate and diagnose glaucomatous OCT reports than healthy OCT reports (Table 2).

**Table 2:** Wilcoxon Rank sum test results for the comparison of the number of fixations and response time between healthy and glaucomatous reports within each expertise group.

Group	Feature	Healthy	Glaucoma
Jr. Resident	# Mean Fixations (value of *p*)	235 (0.324)	236 (0.324)
	Response Time (s)	39.21 (0.271)	39.03 (0.271)
Sr. Resident	# Mean Fixations (value of *p*)	152 (0.0841)	199 (0.0841)
	Response Time (s)	23.82 (0.0668)	32.16 (0.0668)
Expert	# Mean Fixations (value of *p*)	59* (0.00916)	92* (0.00916)
	Response Time (s)	8.187* (0.00366)	13.98* (0.00366)

Expert group demonstrated a significantly greater number of fixations and response times when evaluating glaucomatous OCT reports compared to healthy OCT reports. Resident groups did not exhibit significant differences in the number of fixations, or the time taken to evaluate both types of reports. Note that * indicates significance.

##### 3.1.1.2. Inter-group statistical testing: comparison of number of fixations and response time in OCT reports by diagnosis

The number of fixations and the response times within the healthy and the glaucomatous reports were compared across expertise groups for different permutations (Table 3): jr. resident vs. sr. resident, jr. resident vs. expert, sr. resident vs. expert, jr. and sr. resident vs. expert. Comparisons were made in such a way to identify whether significant differences existed between novice and expert groups for each type of report. It was observed that experts made significantly fewer fixations on healthy reports (59 mean fixations) compared to both jr. and sr. residents (192 mean fixations). The same result was identified for glaucomatous reports: experts made 92 mean fixations compared to jr. residents who made 236 mean fixations, and sr. residents who made 199 mean fixations. Comparisons between jr. and sr. resident fixation occurrence revealed no significant differences in the total number of fixations per report for both healthy and glaucomatous reports between these novice groups.

**Table 3:** Wilcoxon Rank sum test results for the comparison of the total number of fixations in optical coherence tomography (OCT) reports across groups, within healthy and glaucoma reports, separately.

Comparison	Diagnosis	Mean # Fixations (value of *p*)
Jr. Resident vs. Sr. Resident	Healthy	235 vs. 152 (0.450)
	Glaucoma	236 vs. 199 (0.488)
Jr. Resident vs. Expert	Healthy	235 vs. 59* (4.04e-05)
	Glaucoma	236 vs. 92* (0.000254)
Sr. Resident vs. Expert	Healthy	152 vs. 59* (7.40e-05)
	Glaucoma	199 vs. 92* (0.00104)
Jr.& Sr. Resident vs. Expert	Healthy	192 vs. 59* (1.96e-06)
	Glaucoma	217 vs. 92* (4.28e-05)

Note that * indicates significance.

##### 3.1.1.3. Intra-group statistical testing: comparison of number of fixations and duration per region in OCT reports, glaucoma vs. healthy (within group)

**Figure 3:**
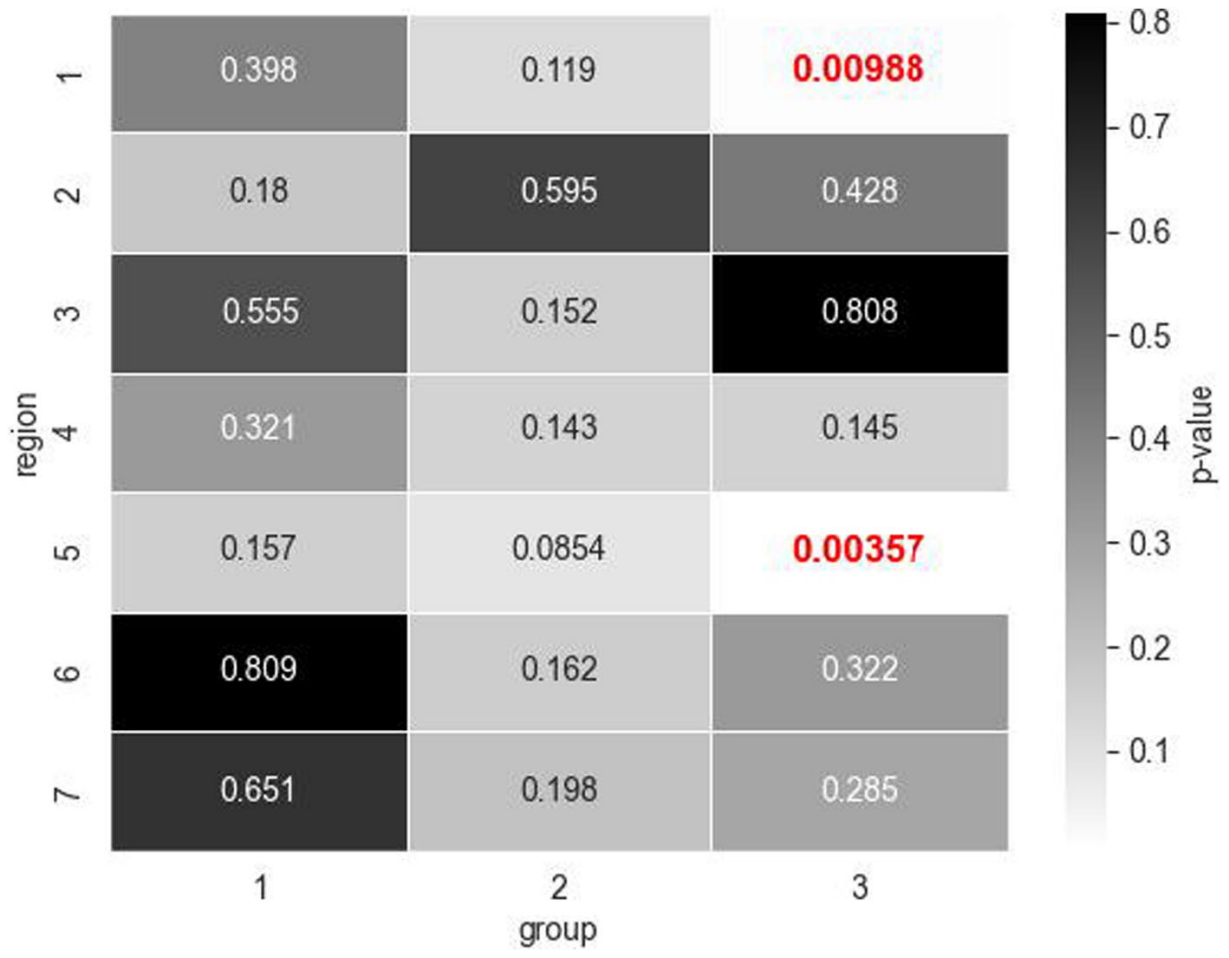
Mann Whitney U test values of *p* for total fixation count comparisons across healthy and glaucomatous OCT reports by regions of interest (ROIs) within expertise groups (1: jr. resident, 2: sr. resident, 3: expert). Expert group exhibited significant differences in the number of fixations for Regions 1 and 5 (values of *p* indicated in red bold font) which correspond to the circumpapillary retinal nerve fiber layer (RNFL) b-scan and the RNFL probability map, respectively.

Based on the statistical tests on the total fixation count and the response time per OCT report, there is an evident difference in the gaze patterns and strategy between the expert and novice groups. Identifying and understanding these differences in search patterns may prove to be critical for informing feature extraction in deep learning model design and for speeding up the learning curve of resident ophthalmologists for glaucoma diagnosis. To further understand what parts of the OCT report play a role in the decision-making process of the expert group at the time of diagnosis, the seven ROIs on the OCT scans that were previously defined were utilized (see Figure 1, right). Statistical testing was performed once more, but this time by region, to evaluate differences in total fixations as a function of the seven OCT report ROIs (Figure 3).

The novice participants’ visual fixation count was similar across the seven regions within both healthy and glaucomatous reports, at a 5% significance level. The expert group, however, displayed significantly more fixations in the circumpapillary RNFL b-scan and RNFL probability map when evaluating glaucomatous reports compared to when evaluating healthy reports. Within the circumpapillary RNFL diagram (Region 1), experts made a mean number of 30 fixations in glaucomatous reports (Table 4A) as opposed to a mean number of 20 fixations in healthy reports (Table 4B). In the RNFL probability map (Region 5), experts made a mean number of 41 fixations for glaucomatous reports as opposed to a mean number of 21 fixations in healthy reports. The Mann–Whitney U statistic was consistent with value of *p*s < 0.01.

**Table 4:** Average number of fixations per region for each training group of participants (A, B) and average duration (in seconds) per region within each training group of participants (C, D) with corresponding percentage of total number of fixations/duration averaged across participants within training group.

(A)	Region 1			Region 5	
Jr. residents:	90, 38.1%			82, 34.8%	
Sr. resident:	74, 37.2%			43, 21.6%	
Experts:	30, 32.6%			41, 44.6%	
	Region 2	Region 3	Region 4	Region 6	Region 7
Jr. residents:	3, 1.27%	9, 3.83%	3, 1.27%	14, 5.93%	35, 14.8%
Sr. residents:	11, 5.53%	18, 9.04%	8, 4.01%	15, 7.52%	30, 15.1%
Experts:	3, 3.26%	5, 5.43%	0, 0%	4, 4.33%	9, 9.78%

(B)	Region 1			Region 5	
Jr. residents:	87, 37.0%			62, 26.4%	
Sr. resident:	62, 40.8%			31, 20.4%	
Experts:	20, 33.9%			21, 35.6%	
	Region 2	Region 3	Region 4	Region 6	Region 7
Jr. residents:	4, 1.70%	12, 5.11%	7, 2.98%	20, 8.51%	43, 18.3%
Sr. residents:	9, 5.92%	14, 9.19%	6, 3.93%	8, 5.26%	22, 14.5%
Experts:	2, 3.38%	5, 8.46%	1, 1.68%	3, 5.08%	7, 11.9%

(C)	Region 1			Region 5	
Jr. residents:	15.1, 38.7%			13.9, 35.6%	
Sr. resident:	12.1, 37.6%			6.91, 21.5%	
Experts:	5.59, 35.9%			6.33. 40.6%	
	Region 2	Region 3	Region 4	Region 6	Region 7
Jr. residents:	0.465, 1.19%	1.31, 3.35%	0.557, 1.44%	2.19, 5.62%	5.53, 14.1%
Sr. residents:	1.84, 5.72%	2.92, 9.08%	1.28, 3.97%	2.39, 7.43%	4.73, 14.7%
Experts:	0.494, 3.17%	0.977, 6.27%	0.102, 0.650%	0.822, 5.27%	1.27, 8.14%

(D)	Region 1			Region 5	
Jr. residents:	14.3, 36.2%			10.4, 26.4%	
Sr. resident:	9.69, 39.8%			4.87, 20.0%	
Experts:	3.39. 36.7%			3.04, 33.0%	
	Region 2	Region 3	Region 4	Region 6	Region 7
Jr. residents:	0.881, 2.24%	1.82, 4.63%	1.08, 2.74%	3.43, 8.69%	7.55, 19.1%
Sr. residents:	2.14, 8.79%	2.20, 9.03%	0.890, 3.66%	1.25, 5.12%	3.31, 13.6%
Experts:	0.277, 3.0%	0.856, 9.28%	0.117, 1.26%	0.536, 5.81%	1.00, 10.85%

Averages and percentages are intentionally categorized by region of interest (ROI) within simplified outline of full OCT report that was presented to participants, **(A)** corresponds to average fixations in glaucoma reports, **(B)** corresponds to average fixations in healthy reports. **(C, D)** Display the average duration (in seconds) per region within each training group of participants, for glaucoma and healthy reports, respectively, with corresponding percentage of mean total duration per report averaged across participants within training group. Note duration in each region also includes time spent on refixations in that region (looking elsewhere and then looking back at that particular region).

The average duration per region within each group (as detailed in Tables 4C,D) further reinforces this observation regarding mean fixation count differences based on regions. The experts allocated more time evaluating Regions 1 and 5 when assessing glaucoma-positive reports compared to healthy ones, spending an average of 5.59 and 6.33 s on these regions, respectively, as opposed to 3.39 and 3.04 s for healthy reports.

##### 3.1.1.4. Inter-group statistical testing: comparison of number of fixations per region in OCT reports, novice vs. expert group

We next determined whether there were particular regions on the healthy and glaucomatous OCT full reports that had a significantly different total number of fixations across the 3 groups with different experience levels. Mann–Whitney U test results revealed that experts had significantly fewer total fixations in Region 1, the circumpapillary RNFL b-scan (glaucoma: mean of 30 fixations compared to a mean of 74 fixations, healthy: mean of 20 fixations compared to a mean of 62 fixations) and in Region 4, the sectoral thickness pie charts (glaucoma: mean of 0 fixations compared to mean of 8 fixations, healthy: mean of 1 fixation compared to a mean of 6 fixations) compared to sr. residents for both glaucomatous and healthy OCT reports. Additionally, sr. residents had significantly fewer total fixations on Region 1, circumpapillary RNFL b-scan (glaucoma: a mean of 74 fixations compared to a mean of 90 fixations, healthy: a mean of 62 fixations compared to a mean of 87 fixations), compared to jr. residents for both diagnoses as well (Table 4). The observed differences in fixation occurrence for these specific regions are of particular interest given their overlap for both healthy and glaucomatous OCT reports, suggesting that sr. residents and experts need fewer fixations overall to make their decisions than jr. residents even though experts fixate significantly more on these regions when distinguishing glaucomatous from healthy OCT reports.

#### 3.1.2. Binary classification using number of fixations: supervised learning approach

**Figure 4:**
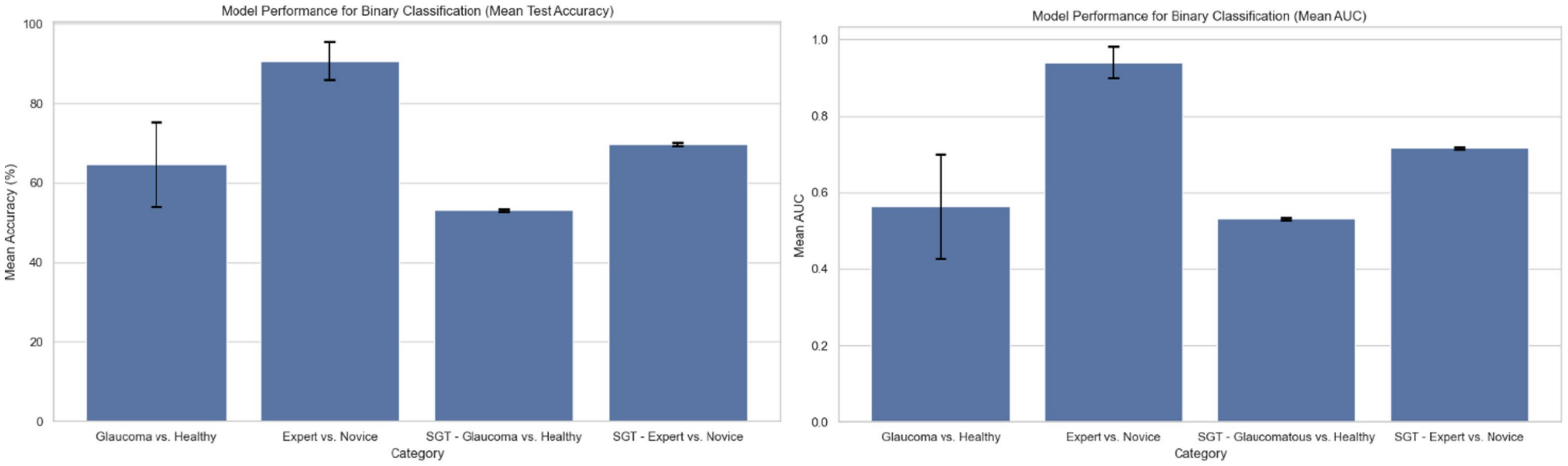
Model performance for binary classification based on region of interest (ROI) fixation positional encoding and based on sequence graph transform (SGT) embedding approach (average test accuracy and AUC based on 50 randomized train-test splits).

The statistical analyses of the total number of fixations across reports and groups confirmed the existence of an underlying difference in the way that experts look at and assess OCT reports to make glaucoma diagnoses. To explore and leverage these differences, we constructed and tested a simple neural network’s ability to discern glaucomatous from healthy reports and experts from novices based purely on the spatial and temporal occurrence of their visual fixations. For the first classification task, the model exhibited a mean test accuracy of 64.67% ± 10.647% and a mean AUC of 0.564 ± 0.137 based on 50 randomized train-test splits (Figure 4). The model’s accuracy is greater than 0.5, so it outperforms mere chance. For the second classification task, the model achieved accuracies greater than 90% for the test dataset and an AUC of 0.94 ± 0.041 (Figure 4), indicating a high accuracy for prediction. Note that due to class imbalance, chance was 62.5% (100 expert samples and 160 novice samples).

### 3.2. Mapping the relationship between fixations and developing a supervised learning model

#### 3.2.1. Unsupervised learning

**Figure 5:**
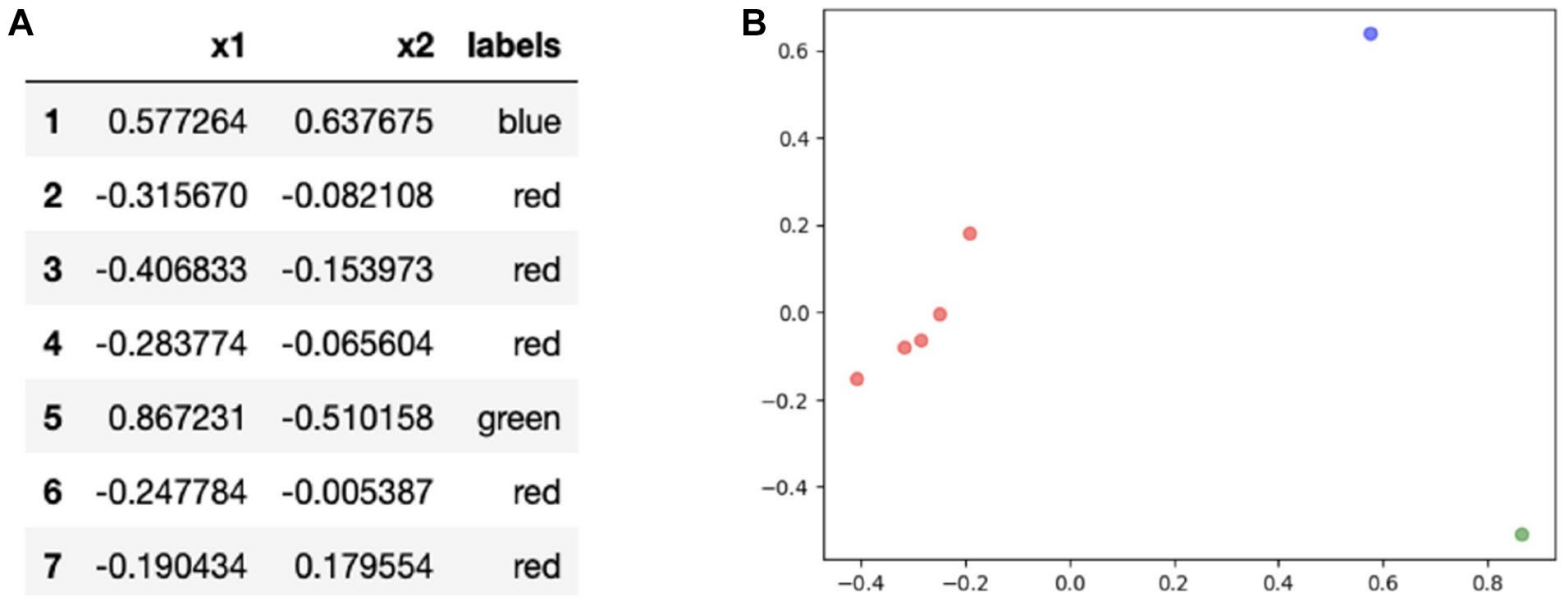
**(A)** Results for principal components analysis (PCA) application on embeddings and **(B)** visualizing the clusters of regions of interest (ROIs) in two dimensions.

**Figure 6:**
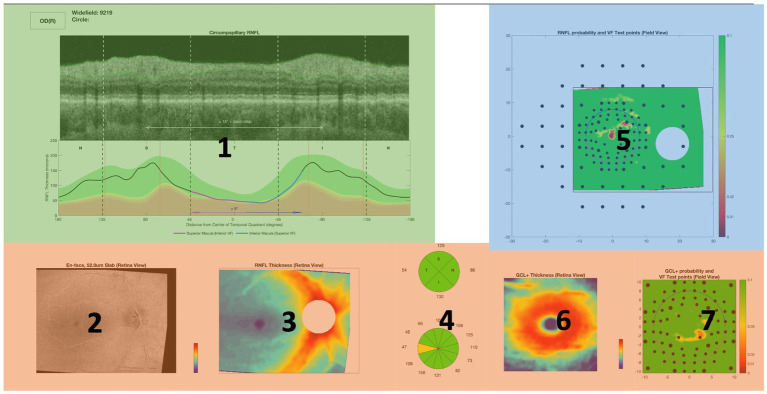
Visual representation of the ROIs contained in each cluster mapped onto an OCT full report. Red cluster contains Regions 2, 3, 4, 6, and 7 while Regions 1 (blue) and 5 (green) were clustered separately.

K-means clustering was performed on each of the OCT Topcon reports for each of the clinicians using the SGT embeddings. A two-dimensional plot visualizing the resulting ROI clusters is shown in Figure 5B. Out of all the reports, one cluster contained Regions 2, 3, 4, 6, and 7 (red) while Regions 1 (blue) and 5 (green) were clustered separately. A visual representation is shown of the clusters in two dimensions, and the clusters mapped onto the OCT Topcon report are shown in Figure 6.

#### 3.2.2. Supervised learning

The results from the unsupervised learning algorithms uncover unique search strategies and patterns across training expertise within both glaucomatous and healthy reports. Based on the embeddings generated, two neural network models were constructed and evaluated to predict either the diagnosis of the report or the level of expertise of clinicians.

#### 3.2.3. Supervised task 1: predicting diagnosis

The first supervised task predicted if the classification of the embeddings corresponded to glaucomatous or healthy OCT reports. The mean training and test accuracies are calculated by training and testing the model with different random train-test splits for 50 iterations. The results are shown in Figure 4. The mean Youden’s J statistic reported was 0.503.

#### 3.2.4. Supervised task 2: predicting expertise group

The mean training and test accuracies for the second supervised task of distinguishing between expert vs. novice OCT-report viewers based on applying SGT embeddings to fixations were also calculated by training and testing the model on different, randomized train-test splits for 50 iterations. The results are shown in Figure 4. The mean Youden’s J statistic was 0.63.

## 4. Discussion

The act of fixating on a particular location provides insight into the cognitive and behavioral components of the viewer’s allocation of attention (32). Our qualitative studies suggest that ophthalmology experts demonstrate refined eye movements, with fewer fixation counts, while performing OCT image screening, compared to ophthalmology residents. This observation is consistent with previous studies comparing the performance of experts versus novices while performing their tasks, including in chess playing (33), sports refereeing (34), and medical image screening. In radiology specifically, eye-tracking research has demonstrated that experienced radiographers made significantly fewer fixations searching for chest nodules on films compared to novice film readers and postgraduate film readers. They not only examined the image in ‘longer, sweeping eye movements’, but also focused on a smaller number of regions for a more detailed inspection and made much better decisions in less time compared to novice observers (35). Interestingly, a previous study that evaluated the visual search pattern of radiologists when evaluating abdomen and pelvic CTs found that attendings made significantly fewer fixations per second than residents, but no significant difference was found in the total number of fixations made by the two groups (36). In contrast, in our ophthalmology glaucoma detection case, the expert vs. novice difference was found to be evident at the total fixation number and at the duration level. This study is one of the first to assess these differences in an ophthalmology setting and the first to evaluate eye movements of clinicians on OCT reports.

### 4.1. Statistical analysis of fixation number across diagnoses and training levels

Expert participants demonstrated fewer total fixations on OCT full reports than novice participants (jr. and sr. residents with <3 years of experience) when diagnosing glaucoma. Additionally, experts spent significantly more time and made significantly more total fixations on the reports when examining reports with glaucoma compared to healthy reports. The region-based statistical analysis comparing the frequency of fixations between healthy and glaucomatous reports across participant data and stratified by training experience further pin-pointed these differences in fixations between experts and novices spatially to the circumpapillary RNFL (Region 1) and the sectoral thickness pie charts (Region 4). Within the expert group, the number of total fixations increased significantly in the circumpapillary RNFL b-scan (Region 1) and RNFL probability map (Region 5), specifically when they reviewed glaucomatous OCT reports. These findings are consistent with the ‘CU method’ that prioritizes analyzing the RNFL probability map first in order to detect glaucoma in OCT reports. If uncertain, the method suggests finding confirmatory evidence in diagrams such as the circumpapillary RNFL b-scan (Region 1) (37).

### 4.2. Unsupervised learning: mapping the relationship between fixations

**Figure 7:**
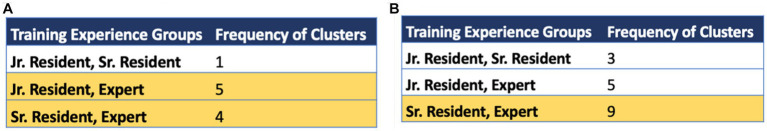
Frequency of same cluster groupings across training expertise groups for **(A)** healthy optical coherence tomography (OCT) reports and **(B)** glaucomatous OCT reports. The rows highlighted in yellow indicate the top 1 or 2 groups with highest common cluster frequencies observed in healthy and glaucomatous reports, respectively. Note that the highest cluster agreement is between sr. residents and experts.

SGT-embedding-based clustering patterns were compared between the different training expertise groups for each of the Topcon reports. There were 10 healthy images shown to each clinician and 10 glaucoma images. We analyzed the clusters for this set of 20 images and found which expertise groups had similar clustering patterns. For example, out of the healthy reports, the sr. residents and experts had similar clustering patterns for 4 unique reports. Figure 7 gives the associated frequency for similar clustering patterns between expertise groups for healthy and glaucomatous reports. For specifically healthy classifications, the most frequent similar cluster pairing was found between the jr. residents and experts. The second most frequent similar cluster pairing was between the sr. residents and experts. For glaucoma classification, the most frequent cluster pairing was found between sr. residents and experts with a total of 9 clusters. This suggests, as we would anticipate as residents gain experience, that sr. residents and experts are using search strategies that have the most in common.

### 4.3. Motivation behind supervised deep learning models

The statistical analysis and unsupervised learning approach provided motivation for the development of supervised deep learning models, as it is evident from the previous sections that they carry information about the decision-making processes of clinicians when diagnosing glaucomatous vs. healthy OCT reports. Due to the lack of statistical significance between report types in the novice group, the input to the supervised model approach that classified healthy vs. glaucomatous OCT reports was restricted to data from the expert group (ophthalmology faculty and fellows).

Both the embedding and fixation number derived supervised approaches resulted in models that distinguish between the eye movements of expert and novice clinicians when diagnosing OCT reports. The unsupervised approach using fixation data found different eye movement patterns between the three different training groups especially for glaucomatous vs. healthy OCT reports. The difference in clusters from the unsupervised embedding approach between the expertise groups for glaucomatous and healthy reports provided motivation for the application of supervised learning to recognize patterns between these groups. Therefore, the fixation-based and embedding-based unsupervised approaches motivated the design of supervised models to detect glaucomatous vs. healthy OCT reports and experts vs. novices from eye movements alone. The results showed that the fixation-based supervised model trained to detect experts vs. novices achieved highest accuracy; such models could serve as a tool in medical education to assess progression/learning of trainees.

### 4.4. Supervised model performance

Each of the models developed explore the features of pupil trajectories and fixations to predict either a diagnosis of glaucoma or a clinician’s expertise level. The variation in model accuracy and AUC values between these models might be due to the number of clinicians within this study. This study contained eye movement data from thirteen clinicians that were collected over the course of 4 months for a total of 260 samples. The model that distinguishes between glaucoma and healthy reports has an input size of 100 samples since the data was restricted to that of expert eye movements. It is likely that the model accuracy will increase with more clinician eye movement data included in the study, as the model would better capture patterns in a larger dataset. Furthermore, addition of dropout, batch normalization, and a custom loss function accounting for class imbalance may prevent overfitting observed especially in our embedding-based supervised model.

Additionally, the trajectory feature samples in the input matrix were cut or extended to 100 fixations per sample based on the median number of fixations across all participants and images. For those trajectories that were cut short, meaningful information on the visual trajectory was lost at the expense of sample size standardization. Similarly, the zero-padding used for the trajectory feature input matrix might have contributed to the model’s accuracy. Since experts made significantly fewer fixations than the jr. and sr. ophthalmology residents, the trajectory feature vectors for these participants were zero-padded in order to standardize the length of the matrix to 100 fixations per sample. The fixation-based expert vs. novice model might have learned the higher content of zeroes in expert positional encoding vectors compared to the novice vectors, explaining its high accuracy at 94.0%.

### 4.5. Analysis of ROIs on OCT reports

#### 4.5.1. Unsupervised learning: analysis of clusters

The most popular clustering found from the unsupervised learning techniques reflects key decision-making strategies used based on the different regions on the OCT report. Based on the clusters (Figure 5), clinicians may have different gaze behaviors in the circumpapillary RNFL b-scan and RNFL probability map since they were clustered separately. The remaining regions are clustered together which suggest similar eye movements among the remaining ROIs.

#### 4.5.2. Intra-group analysis: total fixation count by region

After conducting the region-based statistical analysis, we discovered significant differences in fixation frequency between healthy and glaucomatous reports, with variations based on participants’ training experiences that are consistent with findings reported in past studies (37). This analysis helped us spatially identify areas of fixation differences on OCT reports between experts and novices, specifically in the circumpapillary RNFL b-scan and RNFL probability map. Furthermore, we observed that within the expert group, the total number of fixations significantly increased in the circumpapillary RNFL b-scan and RNFL probability map when they reviewed glaucomatous OCT reports. These findings suggest that experts have a more focused gaze search strategy than novices when reviewing OCT reports, as they fixate more frequently in specific regions. Moreover, the increased fixations in the circumpapillary RNFL and RNFL probability map in glaucomatous OCT reports among experts indicate a heightened awareness of critical features associated with glaucoma diagnosis in these regions.

#### 4.5.3. Supervised models feature importance

**Figure 8:**
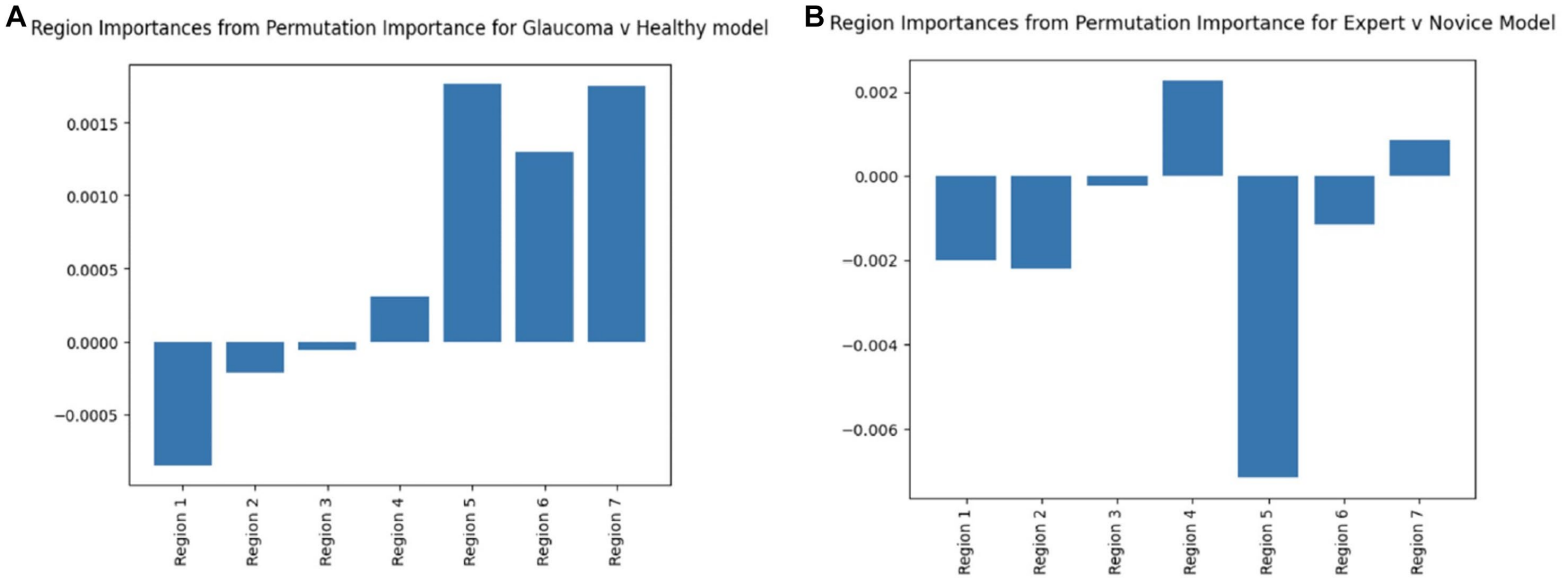
Region permutation importance chart using model weights in test data for **(A)** Glaucoma vs. Healthy model and **(B)** Expert vs. Novice Model. Both models illustrate Region 5 (RNFL probability map) as the most important feature.

The feature importance diagrams (computed via permutation importance: random shuffling of a given feature to evaluate its importance for model classification) for both embedding derived models that classified healthy vs. glaucomatous and expert vs. novice eye fixations, draw attention to regions that were most meaningful in order for the model to learn patterns within different classes. Based on Figure 8, the RNFL probability map (Region 5) has the highest importance in both deep learning models. In the glaucoma vs. healthy model, the GCL + thickness and GCL + probability maps are also among the most important features. These findings further confirm our past work, in which eye movements of just two OCT experts were compared to AI-based concept activation vectors; the same three regions (RNFL and GCL probability and GCL thickness maps) were found to be most important for successful AI and expert glaucoma classification (4).

The significance of both the circumpapillary RNFL b-scan and RNFL probability map in detecting glaucoma is highlighted through the above results. The use of the RNFL probability map in particular by the clinicians is again consistent with the ‘CU method’ adopted by clinicians at Columbia to diagnose glaucoma from OCT reports (37).

### 4.6. Clinician’s accuracy and search strategies

Each of the twenty reports classified by clinicians had ground truth labels of Healthy or Glaucoma (based on prior expert review of clinical and imaging data). To evaluate the accuracy of the thirteen clinicians’ classifications, an average accuracy score was calculated by comparing their diagnoses with the provided labels. The average accuracy of clinicians correctly classifying OCT reports was calculated to be 83.1 ± 5.38%.

This high accuracy score not only serves as a validation of the data collection process but also highlights the limitations of diagnosing glaucoma solely based on an OCT report. Glaucoma is a complex disease that often requires the evaluation of additional factors such as intraocular pressure, visual fields (VFs), and patient history. The agreement across this data enables clinicians to make more accurate diagnoses. It is possible that due to the lack of this additional patient data, incorrect diagnoses were made by clinicians.

The accuracy score was also determined for the two levels of novice participants. For the eight novice clinicians, the mean accuracy was 83.8 ± 4.15%. The mean accuracy for the five expert clinicians was 82.0 ± 6.78%. The similar scores between the experts and the novices show that, in spite of expertise-dependent variations in cognitive decision-making strategies for glaucoma detection, the eye tracking data we used in this study achieved a similar standard of glaucoma-detection accuracy across participants.

The determination of these accuracy scores reveals opportunities to analyze an ophthalmologist’s eye movements on a correct versus incorrect diagnosis. For example, we can evaluate the statistical analysis of fixations and regional clusters from only correctly classified reports and compare them with the results in this study. This may also provide insight into understanding ophthalmologist’s cognitive practices for definitive vs. inconclusive/ambiguous reports. In addition, analyzing eye fixations on reports incorrectly diagnosed by the novice training groups could aid in interpreting and improving the choices made by trainees to ultimately build optimized medical education tools.

### 4.7. Conclusions and future directions

Our analysis of expert eye movements on OCT reports has provided valuable insights into the cognitive diagnostic practices employed by clinicians. The comparison of visual fixation patterns across different expertise groups has identified potential opportunities for the development of medical education/skill assessment tools. Our study aimed to determine if there were significant variations in clinical visual search patterns on OCT reports when diagnosing glaucoma based on expertise. Additionally, we sought to establish whether fixation trajectory and fixations are reliable features to build robust AI models for glaucoma detection. To analyze decision-making strategies, we focused on seven regions of interest (ROIs) in the OCT report and examined the statistical differences in fixation count between each region. Furthermore, we explored the embedding clusters generated by quantifying the relationship between fixations.

Based on the visual fixation features of the gaze data of the thirteen experts who participated in this study, our results suggest the existence of an underlying difference in the search pattern of clinicians when diagnosing glaucoma on OCT reports, as a function of expertise. Our statistical analyses and unsupervised learning approaches provided evidence to indicate that expert ophthalmologists make fewer, refined eye fixations when scanning OCT reports and that the RNFL probability map and the circumpapillary RNFL b-scan are two diagrams that play a fundamental role in the process of glaucoma diagnosis, which is consistent with the ‘CU method’.

Motivated by our unsupervised approaches, we developed and utilized supervised models to distinguish between healthy vs. glaucomatous OCT reports and to differentiate between eye movements of expert vs. novice ophthalmologists. Interestingly, the performance of the model comparing expert vs. novice ophthalmologists outperformed the model trained to distinguish glaucomatous vs. healthy reports. To further enhance the performance of these models, collecting additional eye movement data from experts would be beneficial to improve the accuracy and the reliability of the predictions made by the models. One of the biggest limitations of our study was the variance of the Pupil Labs Core eye tracker. Therefore, the use of higher-frequency eye trackers, such as the Tobii Pro Fusion, may also enhance the performance of these models and our overall understanding of search gaze patterns. A higher sampling rate enables the eye tracker to capture more precise and accurate data such as small changes in eye positions and directions. This more fine-grained data may potentially reveal more distinct gaze patterns between the classification groups, allowing the ML models to make more precise predictions.

It has been discussed in previous studies that experts generally make fewer visual fixations per second within the task of their expertise, but these fixations tend to have longer durations than those of trainees, to allow more time for information extraction (38). Incorporating fixation duration or saccade amplitude/velocity as input features in future studies could provide further insights into glaucoma detection for both understanding the diagnostic process and eventually designing medical education/skill assessment tools for ophthalmology residents.

The techniques and analyses investigated in this study reveal potential opportunities for developing interactive tools for medical education. For example, ophthalmology trainee progress can be assessed via the number of their eye fixations in specified regions as they diagnose OCT reports. Additionally, the sequence of their eye fixations can also be evaluated and compared to expert fixation trajectories on OCT reports for glaucoma detection using existing procedures such as the ‘CU method’. Future directions of this work could also include evaluating eye movements on varying OCT sub-images (e.g., just a magnified circumpapillary RNFL b-scan, RNFL probability map, or key slices from a full OCT b-scan volume). In future work, we aim to extend our data collection, SGT-based feature extraction, and model development pipelines to eye movements of residents and experts from other institutions beyond Columbia to determine if regions of interest and patterns of performance observed across expertise levels remain the same or change as a function of variation in institution (and thus variation in training paradigms).

Overall, this study sought to understand the cognitive strategies used by clinicians for detecting glaucoma; the ultimate goal of this line of inquiry will be the design AI systems that can aid in medical education and glaucoma diagnosis.

## Data availability statement

The raw data supporting the conclusions of this article will be made available by the authors, without undue reservation.

## Ethics statement

The studies involving humans were approved by Columbia University Irving Medical Center Institutional Review Board IRB Protocol # AAAU4079. The studies were conducted in accordance with the local legislation and institutional requirements. The participants provided their written informed consent to participate in this study.

## Author contributions

MA, SC, and KAT contributed to conception and design of the study. MA performed the statistical analysis and designed deep learning approaches. SC analyzed eye fixation relationships through SGT embeddings and developed supervised learning approaches. MA and SC wrote the first draft of the manuscript. JML, GAC, and RWSC reviewed and edited the final manuscript for clinical accuracy. KAT provided guidance and oversight on eye tracking and deep learning approaches as well as final manuscript review at all stages. All authors contributed to the article and approved the submitted version.
