# Supplementary material for: Extracting decision-making features from the unstructured eye movements of clinicians on glaucoma OCT reports and developing AI models to classify expertise
Source: Front Med (Lausanne). 2023 Sep 29;10:1251183. doi: 10.3389/fmed.2023.1251183 (PMC10571140; doi:10.3389/fmed.2023.1251183)
Supplement: Supplementary file 1 [file Image_1.PDF]

Supplementary Material

Extracting Decision-Making Features from the Unstructured Eye Movements of Clinicians on Glaucoma OCT Reports and Developing AI Models to Classify Expertise

Michelle Akerman[1†], Sanmati Choudhary[2†], Jeffrey M. Liebmann[3], George Cioffi[3], Royce W.S. Chen[3], Kaveri A. Thakoor[1,2,3*]

*** Correspondence:** Corresponding Author: k.thakoor@columbia.edu

[†]equal contribution

Supplementary Figure and Table

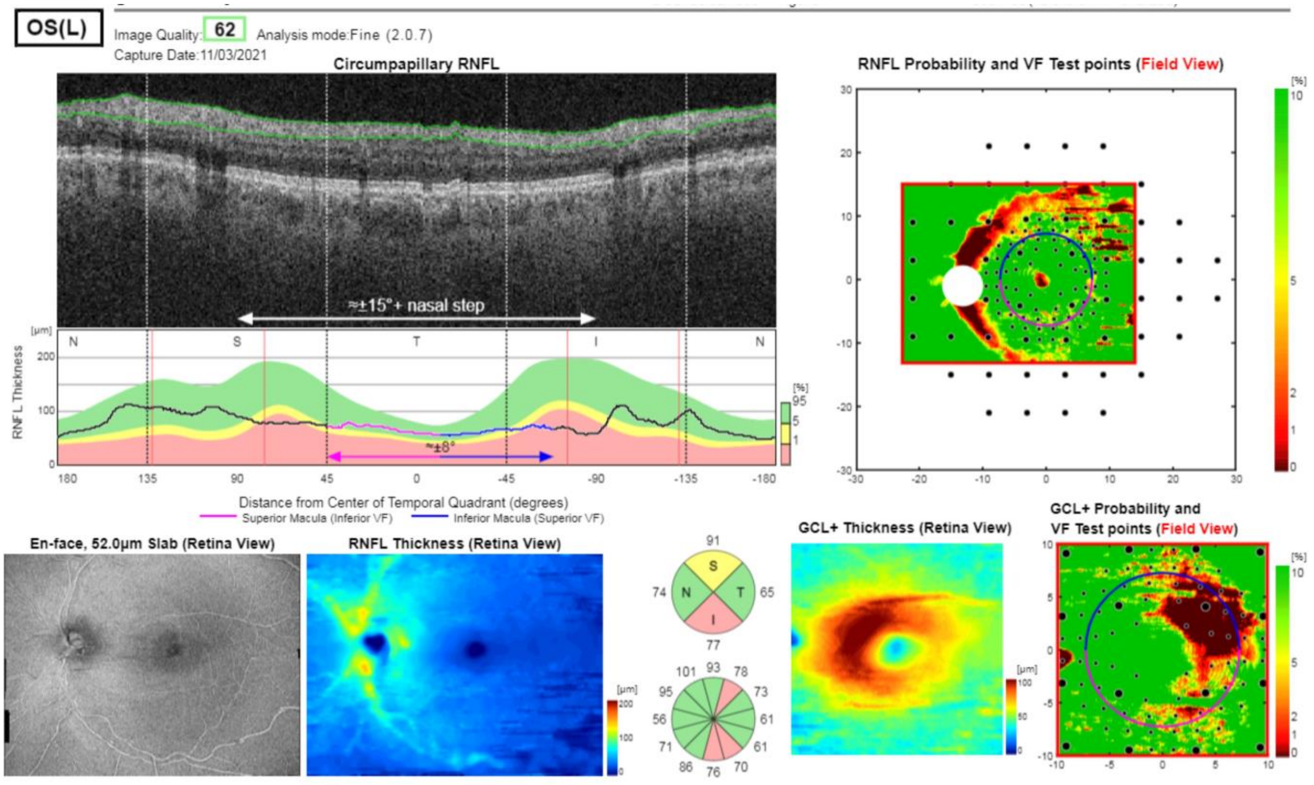

Figure S1. This figure represents an example of a clean, digital Topcon OCT report that was analyzed by each of our thirteen participants.
